# Design and Realization of a Planar Ultrawideband Antenna with Notch Band at 3.5 GHz

**DOI:** 10.1155/2014/563830

**Published:** 2014-07-15

**Authors:** Rezaul Azim, Mohammad Tariqul Islam, Norbahiah Misran, Baharudin Yatim, Haslina Arshad

**Affiliations:** ^1^Departemnt of Physics, University of Chittagong, Chittagong 4331, Bangladesh; ^2^Space Science Centre (ANGKASA), Universiti Kebangsaan Malaysia, 43600 UKM Bangi, Malaysia; ^3^Department of Electrical, Electronic & Systems Engineering, Universiti Kebangsaan Malaysia, 43600 UKM Bangi, Malaysia; ^4^School of Applied Physics, Faculty of Science & Technology, Universiti Kebangsaan Malaysia, 43600 UKM Bangi, Malaysia; ^5^Center of Artificial Intelligent Technology, Faculty of Information Science & Technology, Universiti Kebangsaan Malaysia, 43600 UKM Bangi, Malaysia

## Abstract

A small antenna with single notch band at 3.5 GHz is designed for ultrawideband (UWB) communication applications. The fabricated antenna comprises a radiating monopole element and a perfectly conducting ground plane with a wide slot. To achieve a notch band at 3.5 GHz, a parasitic element has been inserted in the same plane of the substrate along with the radiating patch. Experimental results shows that, by properly adjusting the position of the parasitic element, the designed antenna can achieve an ultrawide operating band of 3.04 to 11 GHz with a notched band operating at 3.31–3.84 GHz. Moreover, the proposed antenna achieved a good gain except at the notched band and exhibits symmetric radiation patterns throughout the operating band. The prototype of the proposed antenna possesses a very compact size and uses simple structures to attain the stop band characteristic with an aim to lessen the interference between UWB and worldwide interoperability for microwave access (WiMAX) band.

## 1. Introduction

In 2002, the federal communications commission (FCC) of the US declared the frequency band of 3.1–10.6 GHz as an open band for ultrawideband (UWB) radio communication [1]. Since then UWB, a high speed wireless communication technology, has recently attracted enormous attention both in industry and in academia. UWB technology is featured with simple, inexpensive, low power consumption and the requirement of a simple hardware configuration as compared to the conventional wireless communication technologies. The advancement in UWB technology has a great impact on telecommunication, wireless sensor networks, high penetrating radars, microwave imaging, and public transport.

Antennas are the indispensable elements of the UWB wireless communication device, and they play a vital part in the advancement of present day communication systems. Modern UWB antennas should feature a small and compact size to be suitable for portable devices. Compared to the 3D antennas [2], planar microstrip antennas designed on the circuit board are a better choice for many wireless communications. Microstrip planar antennas are very appropriate for UWB application because of attractive advantageous features of low profile, inexpensive, light weight, ease of fabrication, and conformability. Moreover, planar antennas can achieve wide operating band and exhibit omnidirectional radiation patterns. Different types of planar antennas with different geometries are widely investigated as they exhibit the fundamental features of UWB technology [3–5]. Different techniques, optimization algorithm, and use of metamaterial have also been reported to design UWB antennas [6–8]. Slot antennas that possess relatively higher magnetic fields are also reported to be very suitable for UWB application since they tend not to couple strongly with nearby objects [9–11].

The UWB system is a high data transmission-rate wireless communication system and operates in the frequencies between 3.1 to 10.6 GHz. However, the narrow band of Worldwide Interoperability for Microwave Access (WiMAX) in 3.3–3.8 GHz band may interfere with the 3.1 to 10.6 GHz band. A traditional technique to suppress the interfering signals is to use a number of bands-stop filters which may increase the system complication and cost. Alternate approach to protect interference caused by the narrow band signal is to use antennas that are able to filter the spectrum of 3.3–3.8 GHz from the UWB frequency band. Design of antennas with the single stop band is therefore necessary.

Several UWB antennas have already been reported with single, multiple, reconfigurable, and tunable notched band/s. A number of techniques have also been reported to realize the band-notch characteristics. The techniques usually that used to realize a dispensed band are etching of slot on the radiating element/ground plane or inserting a tuning stub in the radiator [12, 13]. An alternate technique is to insert parasitic slits/patches and split-ring resonator/s near the patch, ground plane, or feed line to filter out the unwanted band [14–19]. Use of electromagnetic band gap structure is also observed to realize stop band characteristics [20]. For example, a novel antenna structure which proved its successfulness to reject interference in 5-6 GHz band was presented in [14]. In [15], a planar circular ring antenna with dual notched bands was reported. To mitigate the potential interferences with coexisting wireless systems of WiMAX, lower WALN, and upper WLAN, band-notched antennas suitable for UWB applications were proposed in [16]. An UWB antenna with triple band notch characteristics was reported in [17]. The geometry comprises a rectangular patch, a modified partial ground plane, and has an overall dimension of 30 × 22 mm^2^. In [18], a coplanar waveguide- (CPW-) fed circular slot antenna with wide dual band-notched function and frequency reconfigurable characteristic was presented. It was observed that the designed antenna with a dimension of 32 × 24 mm^2^ was able to achieve a wide operating ranging from 2.7–12 GHz with dual notch bands operating at 3.8–5.9 GHz and 7.7–9.2 GHz. For UWB communication, a low profile antenna with the tunable stop band was proposed in [19]. All the above mentioned antennas are relatively large in size and use complex filter structures to create and control notched band/bands. Moreover, use of varactor diodes to reconfigure and tune the notched band/s increases the system complexity.

Despite the good number band notched antennas, only few antennas are reported with the single notched band for WiMAX such as, in [21], a fan shaped antenna with band notched characteristics was proposed for UWB. The antenna comprises fan shaped patch and is fed by CPW transmission line. To achieve band notch characteristics, an arc-shaped slot was inserted into the radiating patch. It was observed that the arc-shaped act as a resonator in achieving notched frequency band and the designed antenna with an overall size of 35 × 30 mm^2^ achieved an ultrawide operating band with a notched band of 3.23–3.93 GHz. In [22] an UWB antenna with single dispensed band was presented. To achieve single notch band at 3.5 GHz, one capacitively loaded loop (CLL) resonator was placed near the microstrip feed line. It was demonstrated that proper selection of the size of CLL resonator, the reported antenna, can achieve the single notched band of 3.25–3.62 GHz due to the strong coupling between the feed line and CLL resonator. An octagon-shaped planar antenna with ultrawide operating band was proposed by Jalil et al. [23]. By inserting a slanted inverted U-shaped slot in the radiating patch, the designed antenna achieved a wide operating band ranging from 2.8–11 GHz with a stop band of 3.3 to 3.6 GHz with an aim to mitigate the interferences between WiMAX and UWB system. However, its performance was not being validated experimentally. In [24], an elliptical UWB antenna with a reconfigurable stop-band for WiMAX was proposed. The desired notched band was achieved by inserting an enhanced open loop resonator on one side of the substrate close to the microstrip feed line. A SMV1405 tuning varactor diode was loaded on the open loop resonator to control the notched frequency band. Though the designed antenna can successfully stop the WiMAX band, it requires a complex system to control the notched band.

In this paper, a printed planar monopole antenna with a notched band to filter out the WiMAX frequency band is presented and fabricated. The antenna comprises a microstrip line-fed radiating element and a ground plane with a wide tapered-shaped slot and is fabricated on commercially available standard dielectric substrate material. To realize a notch band at 3.5 GHz, a parasitic element is placed on the same side of the substrate along with the patch. It is noted that by properly selecting the size and position of the parasitic element, the proposed geometry could achieve UWB operating band with a single notched band covering the WiMAX narrow band. The proposed antenna design is very simple and easy to fabricate and is able to achieve better system performance. The overall size of the proposed single band notch antenna is 22 × 24 mm^2^, which is smaller than the antennas presented in [21–24], designed for the same application. Moreover, it uses very simple filter element only at one side of the substrate which gives it advantages over many reported band notch UWB antennas.

## 2. Design and Optimization

**Figure 1:**
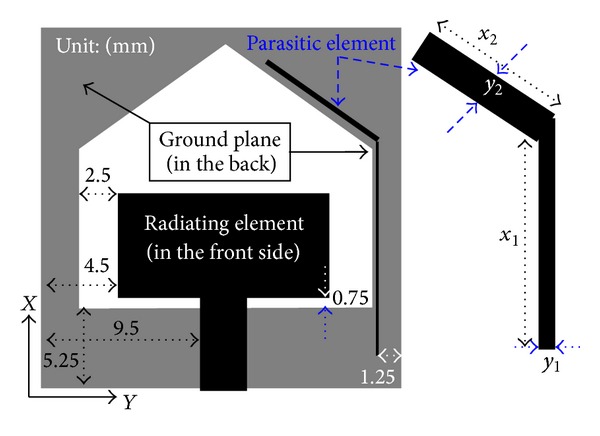
Geometry of the proposed antenna.

The design layout of the single band notch UWB antenna is presented in Figure 1. The reference antenna comprises a radiating element and a ground plane and is fed by microstrip feed line. The radiating element of size 13 × 7 mm^2^ is printed on one side of a substrate while the conducting ground plane with a wide tapered shaped slot is printed on the other side. Commercially available FR4 PCB material with a thickness of 1.6 mm and dielectric constant of 4.6 is used to design the proposed band notch antenna. The 50Ω microstrip fed line has a width and length of 3 mm and 6 mm, respectively, and the gap between the ground plane and patch is* h* = 0.75 mm. It is found that the radiating patch coupled well with the slotted ground and the initial design attained an UWB operating band of 3–11 GHz as presented in [11].

To notch the WiMAX frequency band, a parasitic element (filter element) has been etched on the same side of the substrate along with the radiating element as shown in Figure 1. The parasitic filter element has two parts: one vertical part with length *x*_1_ and width *y*_1_ and one inclined part with length *x*_2_ and width *y*_2_. It is found that the parasitic element couples strongly with radiating element that leads to high impedance at the notch frequency band.

**Figure 2:**
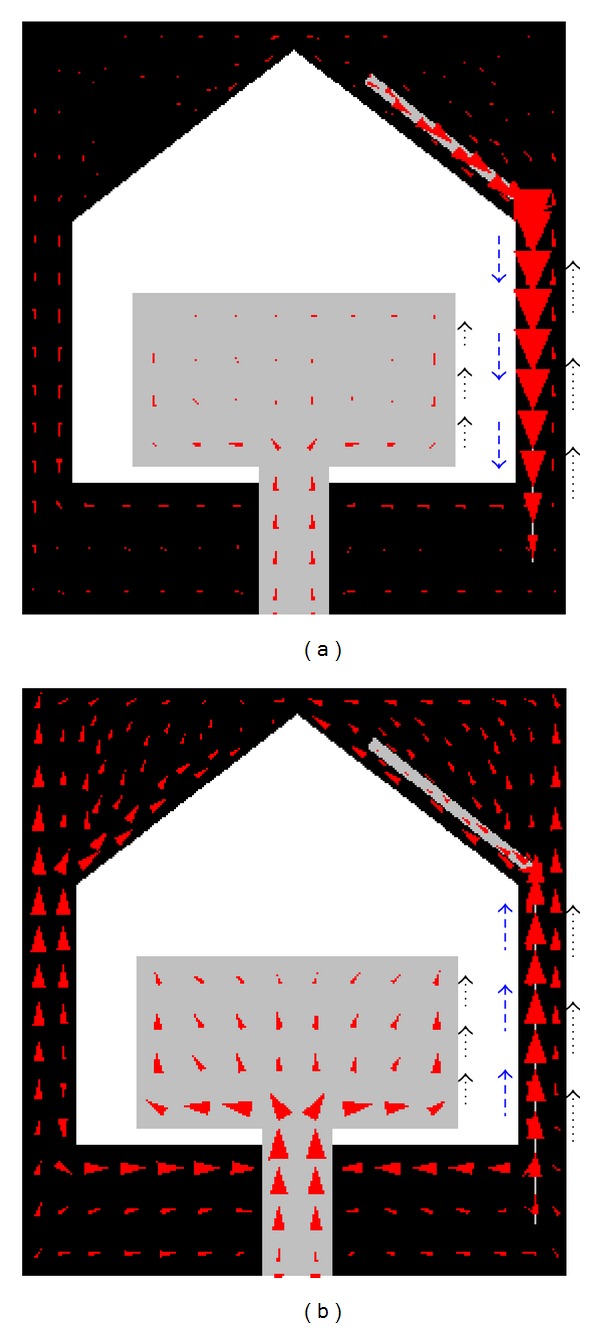
Surface current distribution at (a) 3.5 GHz (notch band) and (b) 5.5 GHz (pass band).

This phenomenon can clearly be understood by examining the surface current distributions. As shown in Figure 2(a), in this structure at 3.5 GHz (notch band), the currents are strongly concentrated around the parasitic element and the flow of the currents in the parasitic element is opposite to that of the ground plane and patch, and they cancel each other as explained in detail in [15, 17]. As a result, the antenna does not radiate effectively at that frequency, and a frequency notch is created around the frequency of 3.5 GHz. At the pass band of 5.5 GHz, the surface current is almost uniformly distributed throughout the radiating patch as well as in the parasitic element and the directions of the current in the parasitic element are the same as that of the patch and the ground plane as depicted in Figure 2(b) resulting in the effective radiation from the antenna.

**Figure 3:**
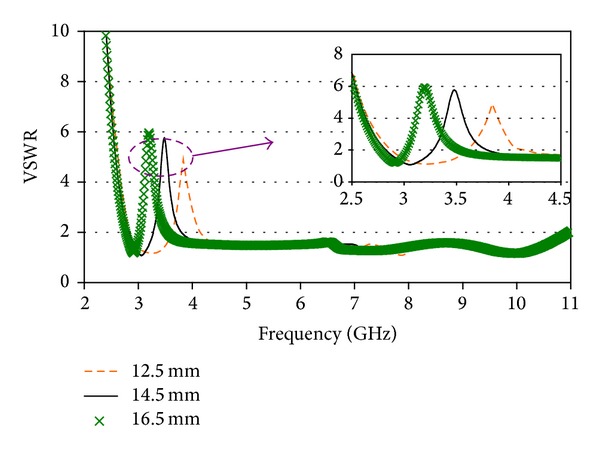
VSWR characteristics for different *x*_1_.

**Figure 4:**
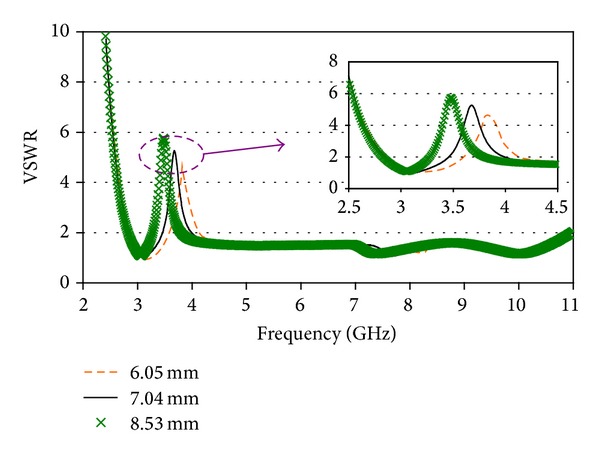
VSWR characteristics for different *x*_2_.

A parametric study has been carried out to examine the effects of different parameters of the filter element on the performance of the proposed antenna including band notched characteristics. Method of moment based simulation software IE3D from Zealand was employed to perform the sensitivity test. Because the etched parasitic element is the only filtering element in realizing the WiMAX stop band, its two key parameters *x*_1_ and *x*_2_ are chosen to analyze their effects on antenna performance. The effects of *x*_1_ and *x*_2_ on antenna performance, including band notch characteristics, are simulated and represented in Figures 3 and 4, respectively. During simulation, all the parameters of the proposed design are kept constant, except the parameter of interest.

The effect of *x*_1_ on VSWR characteristic of the proposed antenna is demonstrated in Figure 3. It is observed from the plot that the bandwidth of the notched band as well as the center frequency is very sensitive to the value of *x*_1_. As the value of *x*_1_ increases from 12.5 mm to 16.5 mm, the notch band moves to the lower frequency, and its bandwidth is getting wider. This is because the increment of *x*_1_ increases the resonating length of the vertical portion of parasitic element resulting in alteration coupling between the parasitic element and patch similar to that explained in [18].

Figure 4 reveals the effect of *x*_2_ on notched band as well as antenna's operating band. It is seen that the WiMAX notched band moves towards the lower frequency band with the increment of *x*_2_. However, the bandwidth of the notched band is reduced with the increment of *x*_2_ and a value of 8.53 mm is optimized to exhibit a stop band centered at 3.5 GHz. Therefore, Figures 3 and 4 show that the 3.5 GHz stop band can be realized and controlled by the etched parasitic element. The figures also demonstrated that when the values of *x*_1_ and *x*_2_ are changed, the SWR characteristics over the entire operating band almost remain unaltered except at the notched band, which offers a great flexibility to choose the filtering element to minimize the interferences between UWB and above mentioned narrow band system. It is revealed that the inclusion of the parasitic element does not change the overall antenna dimension. The optimized parameters of the filter element are as follows: *x*_1_ = 14.5 mm, *x*_2_ = 7.04 mm, *y*_1_ = 0.15 mm, and *y*_2_ = 0.55 mm.

## 3. Experimental Results and Discussion

**Figure 5:**
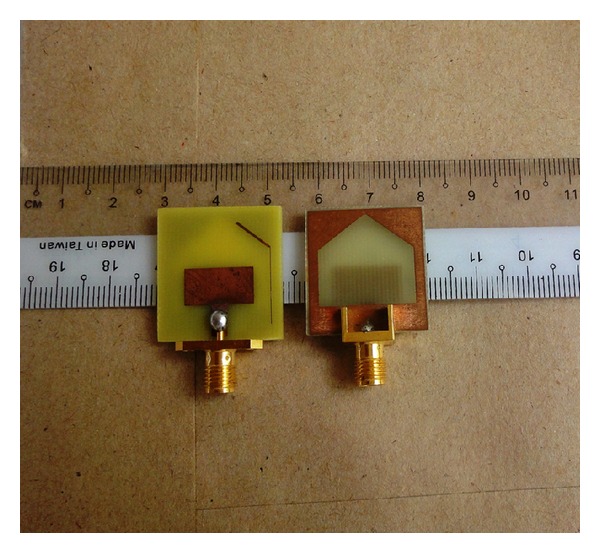
Photograph of the proposed antenna.

**Figure 6:**
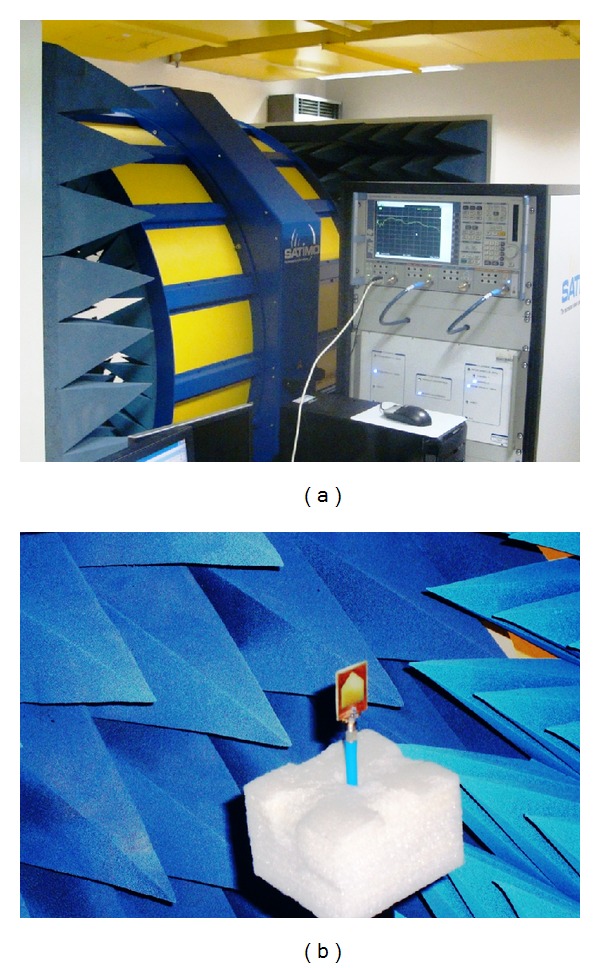
(a) Satimo Starlab antenna measurement system and (b) measurement setup in Satimo Starlab.

With the optimized design parameters, a couple of prototypes was subsequently fabricated for experimental validation and is shown in Figure 5. An Agilent E8362C PNA series vector network analyzer was utilized to measure the SWR characteristics, while a Satimo Starlab 0.6–18 GHz anechoic chamber was used to measure the gain and radiation patterns as shown in Figure 6(a).

The Satimo Starlab system uses the near-field measurement techniques that allow measurement of electric fields within the near-field of the antenna to calculate the equivalent far-field data of the antenna under test [25, 26]. The fabricated antenna is placed on the test board which is located at the center of a circular “arch” that contains 16 probes as shown in Figure 6(b). These probes are situated at an equal distance along the circular surface of the Starlab. The antenna is rotated horizontally through 360°, and the combination of this rotation and the array of probes permits a full three-dimensional scan of the antenna under test, allowing complete radiation patterns to be measured, plotted, and analyzed. Using the measured far-field radiation pattern data, the gain of the antenna under test can then be calculated.

**Figure 7:**
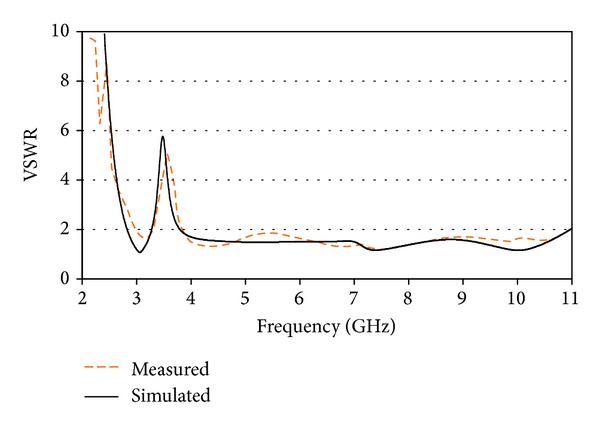
VSWR curves of the proposed antenna.

A comparison of the measured and simulated VSWRs of the proposed antenna is plotted in Figure 7. It is clearly observed from the SWR characteristics that a good agreement between the measured and simulated results has been achieved. The measured result demonstrated that the proposed band notch antenna is able to achieve an operating bandwidth (VSWR ≤ 2) ranging from 3.04 GHz to 11 GHz with a notched band centered at 3.5 GHz (from 3.31–3.84 GHz). Despite its smaller size than the antennas reported in [14, 15, 17, 18, 21–24], the fabricated antenna could achieve UWB operating band with single notch band for WiMAX. The slight disagreement between two VSWR curves is mainly due to fabrication inaccuracies and tolerance of dielectric loss of the substrate. Using the achieved notched band of the proposed antenna, potential inference caused from WiMAX can therefore be avoided completely.

**Figure 8:**
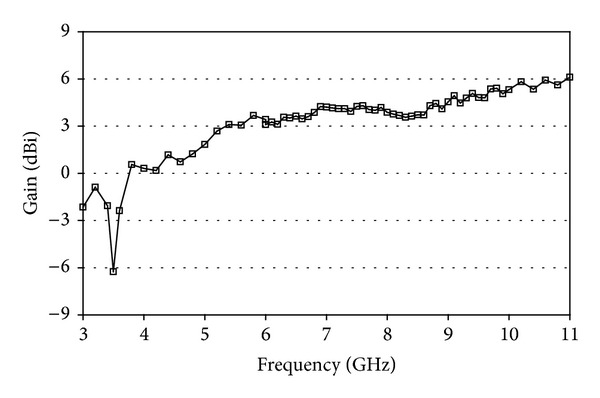
Measured peak gain of the proposed antenna.

Figure 8 illustrates the measured peak gain of the proposed band notch antenna from where it is seen that fabricated antenna achieved an average gain of 3.44 dBi except at notched band. The measured gain at the WiMAX notched band is −6.3 dBi, which is substantially lower than the average antenna gain of 3.44 dBi. A gain of −6.3 dBi is enough to reject the WiMAX frequency band and due to this low gain the proposed antenna is not able to radiate effectively resulting in the creation notched frequency band.

**Figure 9:**
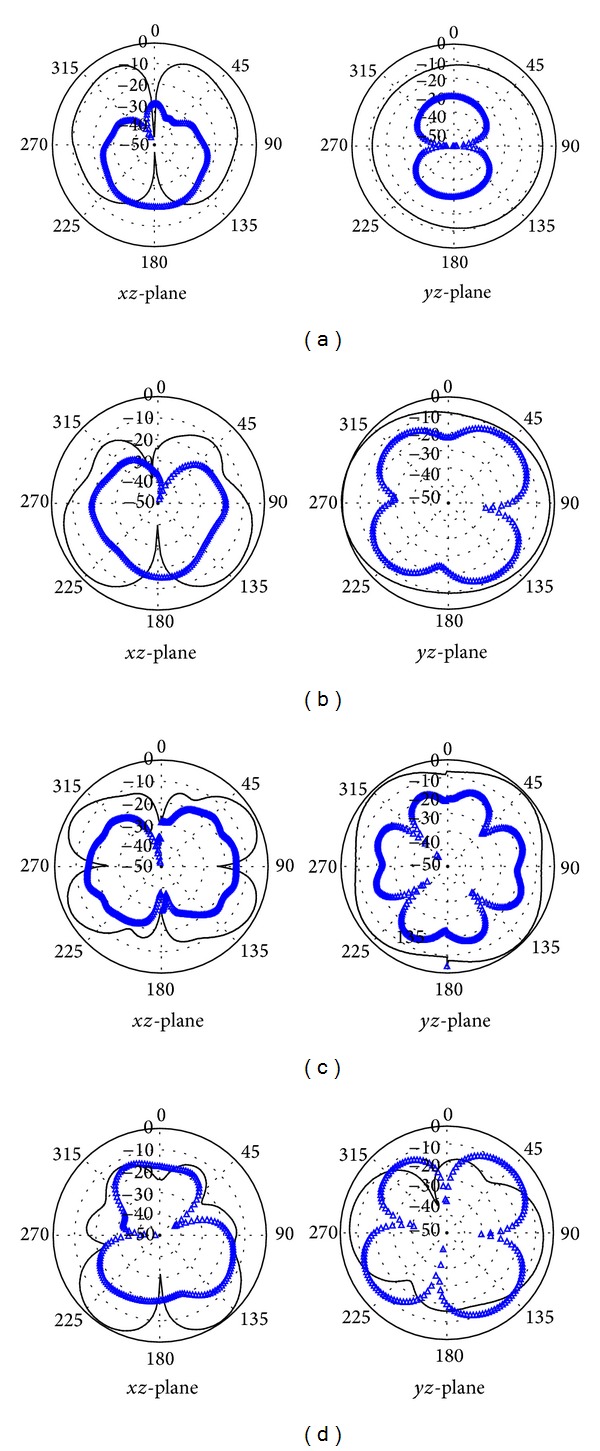
Measured 2D radiation patterns at (a) 3.04 GHz, (b) 6 GHz, (c) 7.38 GHz, and (d) 10 GHz (black solid line: *E*_*θ*_; blue cross line: *E*_*φ*_).

Two-dimensional radiation patters of the realized prototype have been measured at 3.04 GHz, 6 GHz, 7.38 GHz, and 10 GHz and are displayed in Figure 9. It is demonstrated in the plot that, at low frequencies, the copolarized filed (*E*_*θ*_) in *yz*-plane is omnidirectional whereas in *xz*-plane nulls have been observed in the broadside direction and patterns are approximately similar to that of typical monopole antennas. At lower frequencies in both planes, the cross-polarization field (*E*_*φ*_) is reasonably lower than the *E*_*θ*_. However, at higher frequencies in both planes slightly directional radiation patterns have been observed. This can be due to the radiation from inserted filter element and higher order current modes. Despite slightly directional radiation patterns at higher frequencies, the proposed antenna exhibits good radiation characteristics that are very necessary for short distance communication.

**Figure 10:**
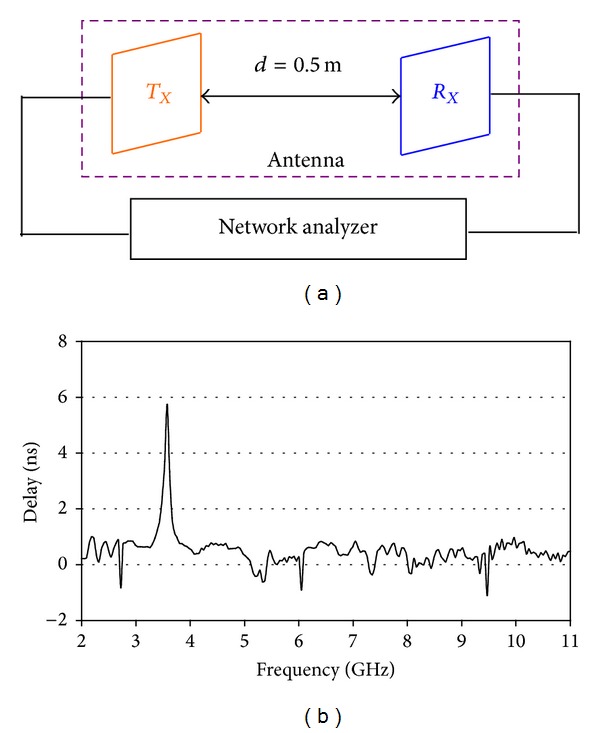
(a) Measurement setup and (b) measured group delay.

Since the UWB antenna directly transmits narrow pulses, its time domain behavior is very crucial. In the high data rate wireless communication system, phase linearity and small group delay are a primary requirement. For a pair of transmitting and receiving antenna system as shown in Figure 10(a), the transfer function (*S*_21_-parameter) is required to have nearly same magnitude and linear phase response over the operating band is required to minimize the distortions in the received signal [26–28]. The group delay is defined as the negative derivative of the phase response. Mathematically

(1) $$\begin{array}{c} \text{Group delay }=-\frac{\Delta S_{21}^{}∠}{\Delta f}, \end{array}$$

where *S*_21_ *∠* is the unwrapped phase response of the transfer function. To analyze the mutual coupling between the antennas, group delay can be used. In UWB system, group delay characterizes the distortion of the signal and it should be the same for all frequencies throughout the operating band. The group delay characteristics between a pair of fabricated antennas had been measured in Satimo's Starlab. In the measurement, the distance between the transmitting (*T*_*X*_) and receiving (*R*_*X*_) antennas is 0.5 m and is placed as face to face orientation as illustrated in Figure 10(a). The measured group delay of the proposed band notch antenna is presented in Figure 10(b). It is observed that the group delay is nearly constant with an average of 0.42 nanoseconds (nS) except at the notch frequency band. At the center frequency of the notched band the delay jumps to 5.74 nS. This small variation in the group delay implies that the designed antenna exhibits good phase linearity at entire UWB band except at notched band.

## 4. Conclusions

A low profile UWB antenna with a notched band centered at 3.5 GHz is proposed and fabricated. To achieve the desired stop band in order to minimize the potential interference between WiMAX and UWB systems, a parasitic element has been introduced on one side of the substrate along with the radiating element. It has been observed that, at notched band, most of the current concentrates around the filter element are in the opposite direction to that of patch and ground plane resulting in the creation of a notched band. It is found experimentally that the proposed antenna with an overall dimension of 22 × 24 mm^2^ is able to achieve an UWB operating band with a notched band of 3.31–3.84 GHz. The designed antenna also achieved a good gain except at notched band and exhibits omnidirectional radiation patterns. The features such as simple structure, inexpensive, low profile, and ultrawide operating band make the proposed antenna appropriate to be used in different wireless communication.
